# Humanised IgG1 antibody variants targeting membrane-bound carcinoembryonic antigen by antibody-dependent cellular cytotoxicity and phagocytosis

**DOI:** 10.1038/sj.bjc.6605355

**Published:** 2009-11-10

**Authors:** S Q Ashraf, P Umana, E Mössner, T Ntouroupi, P Brünker, C Schmidt, J L Wilding, N J Mortensen, W F Bodmer

**Affiliations:** 1 Cancer and Immunogenetics Laboratory, Department of Medical Oncology, Weatherall Institute of Molecular Medicine, Oxford, UK; 2 Department of Colorectal Surgery, John Radcliffe Hospital, Headington, Oxford, UK; 3 Glycart Biotechnology AG, Wagistrasse, Schlieren, Zurich, Switzerland

**Keywords:** PR1A3, CEA, ADCC, ADCP, colorectal cancer, glycoengineering, NK cells, monocyte-derived macrophages

## Abstract

**Background:**

The effect of glycoengineering a membrane specific anti-carcinoembryonic antigen (CEA) (this paper uses the original term CEA for the formally designated CEACAM5) antibody (PR1A3) on its ability to enhance killing of colorectal cancer (CRC) cell lines by human immune effector cells was assessed. *In vivo* efficacy of the antibody was also tested.

**Methods:**

The antibody was modified using EBNA cells cotransfected with *β*-1,4-*N*-acetylglucosaminyltransferase III and the humanised hPR1A3 antibody genes.

**Results:**

The resulting alteration of the Fc segment glycosylation pattern enhances the antibody's binding affinity to the Fc*γ*RIIIa receptor on human immune effector cells but does not alter the antibody's binding capacity. Antibody-dependent cellular cytotoxicity (ADCC) is inhibited in the presence of anti-Fc*γ*RIII blocking antibodies. This glycovariant of hPR1A3 enhances ADCC 10-fold relative to the parent unmodified antibody using either unfractionated peripheral blood mononuclear or natural killer (NK) cells and CEA-positive CRC cells as targets. NK cells are far more potent in eliciting ADCC than either freshly isolated monocytes or granulocytes. Flow cytometry and automated fluorescent microscopy have been used to show that both versions of hPR1A3 can induce antibody-dependent cellular phagocytosis (ADCP) by monocyte-derived macrophages. However, the glycovariant antibody did not mediate enhanced ADCP. This may be explained by the relatively low expression of Fc*γ*RIIIa on cultured macrophages. *In vivo* studies show the efficacy of glycoengineered humanised IgG1 PR1A3 in significantly improving survival in a CRC metastatic murine model.

**Conclusion:**

The greatly enhanced *in vitro* ADCC activity of the glycoengineered version of hPR1A3 is likely to be clinically beneficial.

Antibodies are increasingly used for immunotherapy of cancers, with over 200 currently in trials and 12 licensed for use in treatment (Adams and Weiner, 2005; Carter, 2006; Reichert and Valge-Archer, 2007). There are now three antibodies approved by the US FDA for treatment of advanced colorectal cancer (CRC) (Goldberg, 2005; Saltz *et al*, 2006; Reichert and Valge-Archer, 2007). The main potential advantages of this form of therapy lie in their specificity, lesser side-effects and their ability to elicit a tumour response by multiple mechanisms (Carter, 2006; Reichert and Valge-Archer, 2007). The outcomes of early clinical trials using murine unconjugated and radioconjugated monoclonal antibodies for cancer treatment were disappointing (Goldenberg, 2002; Reichert and Valge-Archer, 2007) probably because of immune responses to foreign proteins reducing serum half-lives, and the delivery of inadequate radiation doses from the radioconjugates to the tumour (Goldenberg, 2002; Sharkey and Goldenberg, 2005; Reichert and Valge-Archer, 2007). Responses were also severely limited by the attenuated ability of the Fc segment of murine antibodies to interact with the relevant receptors of the human immune system. These problems have now been largely overcome by the development of technologies for the production of chimeric, humanised and completely human antibodies (Clynes, 2006).

Two lines of evidence support the importance of immune function for naked antibody therapy. Firstly, Clynes *et al* (2000) showed that antibody-based killing of xenografted and syngeneic tumours was abrogated in Fc*γ*R knockout mice. This established that the ability of antibodies to reduce tumour burden was greatly reduced in the absence of immune cells expressing Fc*γ*Rs. Secondly, colorectal, lymphoma and breast cancer patients carrying the polymorphic variants in the Fc*γ*RIIIa and Fc*γ*RIIa genes that encode for high affinity binding receptors for the Fc antibody segments, have the best clinical outcomes following antibody treatment (Weng and Levy, 2003; Zhang *et al*, 2007; Musolino *et al*, 2008).

Responses to antibody therapy for solid cancers are so far modest for cetuximab and trastuzumab monotherapy. Although cetuximab in combination with irinotecan improves the response rate from 10% to over 20% (Cunningham *et al*, 2004), monotherapy response rates in haematological malignancies are markedly higher than for solid cancers (McLaughlin *et al*, 1998). This may be due to the increased surface area of haematological tumours that is exposed to antibody binding and to NK cells (Olszewski and Grossbard, 2004). Emphasis is now being placed on techniques that ‘tune’ antibodies to make them more efficient at harnessing the immune system for killing solid cancers. Thus, technologies have been developed that engineer Fc antibody segments to enhance their ability to elicit immune effector functions, such as antibody-dependent cellular cytotoxicity (ADCC). One approach uses protein engineering to modify the Fc segment (Lazar *et al*, 2006). Another uses glycoform engineering, which has been shown to improve *in vitro* ADCC by 10–100-fold (Umana *et al*, 1999; Niwa *et al*, 2004b; Schuster *et al*, 2005). In this study, we have used glycoengineering to enhance the immune effector function of an antibody that we have shown earlier to kill CRC-derived cell lines by ADCC (Conaghan *et al*, 2008).

The antibodies now used for the treatment of CRC target EGFR and VEGF (Cunningham *et al*, 2004; Adams and Weiner, 2005; Goldberg, 2005; Carter, 2006; Saltz *et al*, 2006) and only appear to be effective in subsets of patients. Earlier work from our laboratory has shown that the antibody PR1A3, which is specific for membrane-bound carcinoembryonic antigen (CEA, formally designated CEACAM5), elicits ADCC against CEA-positive CRC cell lines even in the presence of super-physiological levels of free CEA (Durbin and Bodmer, 1987; Durbin *et al*, 1994; Conaghan *et al*, 2008). CEA is present on a high proportion of colorectal cancers, in which it is accessible to intravenously administered antibody, whereas in normal colorectal epithelium, due to the apical localisation of the antigen, it is not (Granowska, 1993). Radioconjugates targeting CEA have been used in early phase clinical trials in CRC (Wong *et al*, 2004; Liersch *et al*, 2005), but there is so far no unconjugated anti-CEA antibody licensed for treatment (Blumenthal *et al*, 2008). The pre-clinical studies reported here are directed at optimising PR1A3 for use in the treatment of colorectal cancer. This antibody has been shown to bind specifically to CEA in the B3-GPI domains. This epitope is at the point CEA anchors into the membrane, and this presumably explains why PR1A3 binds to membrane-bound CEA but not soluble CEA (Durbin *et al*, 1994; Conaghan *et al*, 2008).

The three classes of Fc*γ*Rs are expressed on NK cells, known to be potent activators of ADCC (Schmitz *et al*, 2002; Lazar *et al*, 2006; Conaghan *et al*, 2008), and on monocytes and granulocytes, which have also been identified as having at least some potential for antibody-dependent killing of target cells, for example, by antibody-dependent cellular phagocytosis (ADCP) (Munn *et al*, 1991; Huls *et al*, 1999; Dhodapkar *et al*, 2005; Lazar *et al*, 2006; Karagiannis *et al*, 2007; McEarchern *et al*, 2007). We describe here a modified phagocytosis assay, based on that developed by Munn *et al* (1991), and its application to the *in vitro* testing of the ability of humanised and glycoengineered PR1A3 to kill CRC-derived cell lines. We also present *in vivo* data that shows the efficacy of PR1A3 as a therapeutic agent in a mouse xenograft model.

## Materials and methods

### Cell lines

The SKCO-1 (from ATCC), LS174T (from BH Tom, NW University, Chicago), LoVo (from ATCC), HCT116 (from ATCC) and MKN45 (from Cell Services LIF, CRUK, London, UK) cell lines were cultured as described (Conaghan *et al*, 2008). For cytotoxicity and phagocytosis assays, cells were suspended in 2% RPMI 1640 medium with 1% glutamine and 10% FCS (RPMI complete medium).

### Antibodies

#### PR1A3

The original murine IgG1*κ* monoclonal antibody to CEA (Richman and Bodmer, 1987) was humanized by Stewart *et al* (1999). Murine (mPR1A3, IgG1) and unmodified humanized (uhPR1A3, IgG1) antibodies were acquired from the Biotherapeutics Development Unit, Clare Hall, CRUK, London, UK. Murine IgG2a PR1A3 (mPR1A3, IgG2a) and a glycoengineered version of re-derived humanised IgG1 PR1A3 (ghPRA1A3, IgG1) were made by joining the variable region of PR1A3 to murine IgG2a and the complementarity determining regions of PR1A3 to human IgG1 variable regions, respectively (Jones *et al*, 1986). Glycoengineering was as described (Umana *et al*, 1999; Schuster *et al*, 2005). Briefly, vectors containing IgG heavy and light chains, and *β*(1,4)-*N*-acetylglucosaminyltransferase-III (GnT-III), were transfected into EBNA cells. The enzyme modifies the N-glycosylation pattern at Asn-297 of the IgG heavy chain resulting in a high degree of bisected, afucosylated oligosaccharides (Umana *et al*, 1999; Schuster *et al*, 2005; Ferrara *et al*, 2006), which has been shown earlier to increase the affinity to Fc*γ*RIIIa (Ferrara *et al*, 2006). The modified antibody was purified from culture supernatants using Protein-A, cation-exchange chromatography and subsequent size-exclusion chromatography.

#### SM3E

This is a humanised IgG1 antibody that binds with extremely high affinity (*K*_d_=20 pM) to both soluble and membrane-bound CEA (Graff *et al*, 2004). A glycoengineered variant was generated using the methods described above.

#### Blocking antibodies

Purified IgG1 anti-CD16 (Fc*γ*III) (clone 3G8, which is unable to distinguish between Fc*γ*RIIIa and Fc*γ*RIIIb), purified IgG1 anti-CD64 (clone 10.1), and Fab_2_ fragments targeting CD16 (clone 3G8), CD32 (Fc*γ*II, clone 7.3) and CD64 (Fc*γ*II, clone 10.1) were purchased from Ancell Corp, Bayport, MN, USA. Purified IgG2b anti-CD32 (clone IV.3) was purchased from Stemcell Technologies (Vancouver, Canada).

FITC-conjugated murine anti-human IgG (clone G18-145), CD3-FITC, CD11b-PE, CD14-PE, CD15-FITC, CD16-PE, CD19-PE, CD32-PE, CD45-PE, CD56-APC, CD64-FITC, IgG1-FITC (isotype control), IgG1-PE (isotype control) and IgG1-APC (isotype control) were obtained from BD Biosciences, Oxford, UK. Anti-HLA class II (DA-2) was obtained from Monoclonal Antibody Services, CRUK (Brodsky *et al*, 1979).

### Isolation of immune effector cells

Peripheral blood mononuclear cells (PBMCs) were isolated from healthy laboratory volunteers, having taken informed consent, or from buffy coats obtained from single donors (National Blood Service, Bristol, UK). The Rosettesep Human NK isolation cocktail (Stemcell Technologies) was used to obtain an enriched population of human NK cells from blood, as described (Roda *et al*, 2006). Granulocytes were separated using One-step Polymorph (Accurate Chemical and Scientific Corp, Westbury, NY, USA). Monocytes were enriched from blood using the Rosettesep Human Monocyte Enrichment Cocktail (Stemcell Technologies). Purification was verified by phenotypic analysis of surface markers (See Supplementary Materials for details).

### Flow cytometric analysis to compare uhPR1A3 and ghPR1A3 binding to membrane-bound CEA

Cells from SKCO-1, a high CEA expressing CRC line used as target, were incubated in varying concentrations of uhPR1A3 or ghPR1A3 (0.001–100 *μ*g ml^−1^), and with anti-human IgG-FITC as secondary antibody. The cells were then washed once with FACS buffer (PBSA, 1% FCS, 1% Sodium Azide) and the supernatant removed before resuspending in 2% paraformaldehyde in PBSA. FACS analysis was carried out using a FACS Calibur Flow cytometer.

### Fluorescence-based EuTDA cytotoxicity assay

As described (Conaghan *et al*, 2008), a fluorescent probe, BATDA (Blomberg *et al*, 1996) (Perkin Elmer, Boston, MA, USA), was used to label target cells. PBMCs, NK cells, monocytes and granulocytes were used as effectors with varying effector : target cell ratios and concentrations of antibodies. After appropriate incubation, supernatant was added to Europium in 96-well plates and the resulting fluorescence read in a time-resolved fluorometer.

The EuTDA assay was used to compare the ADCC activity of mPRA13 IgG1, mPR1A3 IgG2a and uhPR1A3 IgG1 on SKCO-1 cells, using antibody at a final concentration of 20 *μ*g ml^−1^ and either PBMCs or NK cells as effectors.

The effect of blocking Fc*γ*Rs I, II and III using Fab_2_ or IgG antibodies was assessed using the EuTDA to measure uhPR1A3 mediated ADCC of SKCO-1. PBMC and enriched NK cells were used as effectors. Murine IgG prostate membrane specific antigen (PMSA, provided by Robert Vessella, University of Washington) was used as an isotype murine control.

The relative ability of PBMCs, NK cells, monocytes and granulocytes, enriched, as described earlier, to elicit ADCC was assessed using the EuTDA assay with uhPR1A3.

### Monocyte-derived macrophages for phagocytosis assays

Enriched monocytes were cultured on Lumox Petraperm Plates (Greiner, Bio-One, North America, Monroe, NC, USA), for 8–12 days at 37° in a 5% CO_2_ incubator, in X-VIVO 15 medium (BioWhittaker, Walkersville, MD, USA), supplemented with M-CSF, GM-CSF and *γ*IFN (Peprotech, UK) (see Supplementary Materials for details). Target cells (SKCO-1) were labelled with CellTracker Green CMFDA (Invitrogen, San Diego, CA, USA). Free label was washed off before incubation with varying concentrations of uhPR1A3 or ghPR1A3 (0.1 –10 *μ*g ml^−1^). Effector monocyte-derived macrophages were added (3 : 1–8 : 1 effector : target ratio), the cells were incubated for 1 h at 37° and analysed using FACS Calibur to measure ADCP.

An alternative ADCP assay was developed using an automated microscope (Ikoniscope, Ikonisys, New Haven, CT, USA) (Ntouroupi *et al*, 2008). Target cells and macrophages, stained as described above, were deposited on to a poly-l-lysine coated slide, fixed with 2% formaldehyde. The slides were washed with PBS containing Tween (PBS-Tween), covered with blocking solution and Goat anti-mouse–HRP (1 : 100 dilution in blocking solution) added as a secondary antibody. The slides were washed with PBS-Tween, before adding Tyramide-647 (1 : 100 dilution).

Phagocytosis events were detected using the Ikoniscope imaging system (Ntouroupi *et al*, 2008). Slides are first scanned at low magnification ( × 10) to detect green label and then revisited at high magnification ( × 100) to identify red labelled macrophages with ‘engulfed’ green targets.

### *In vivo* testing of PR1A3

Six to 12-week-old SCID/Beige mice were used for animal experiments. All experiments were performed after ethical approval from the Swiss Veterinary Office. LS174T cells were maintained in DMEM medium with 1% glutamine and 10% FCS (E4 complete medium). A murine model for CRC tumours was set up by intra-splenic injection of LS174T (3 × 10^6^ cells per mouse) under aseptic conditions (day 0). This resulted in the development of liver metastasis. Passive antibody protection was measured by comparing the survival of mice (*n*=10 for each group) after intravenous injection of either glycoengineered humanised IgG1 PR1A3, or glycoengineered high affinity IgG1 anti-CEA antibody (clone SM3E,) or vehicle PBS control. The SM3E antibody, unlike PR1A3, is not membrane CEA specific. Antibody injections were performed at days 7, 14 and 21 with a treatment dose of 25 mg kg^−1^ of bodyweight of mouse. Termination criteria were in accordance with the Swiss Veterinary Office.

### ADCC and ADCP data analysis

Percentage cell lysis in the cytotoxicity assays was calculated as [experimental release−background release]/[maximum release−background release] × 100. Antibody-dependent (specific) lysis was calculated as [experimental release−antibody-independent release]/[maximum release−antibody-independent release] × 100. The standard error of the mean of multiple experiments was calculated using Graphpad Prism software, San Diego, CA, USA. Percentage cell phagocytosis was calculated using the formula: number of dual-stain positive target cells (cells engulfed by macrophages) divided by the total number of target cells. Standard normal distribution tests were performed to test the significance of differences found.

## Results

**Figure 1:**
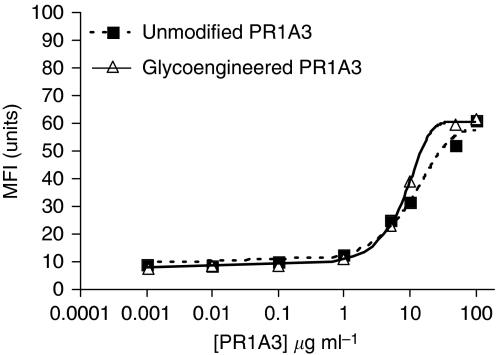
Comparison of the binding of unmodified humanised IgG1 PR1A3 (uhPR1A3) and glycoengineered IgG1 PR1A3 (ghPR1A3) to the high CEA expressing cell line SKCO-1. Mean fluorescent intensities, based on flow cytometric analysis, of uhPR1A3 and ghPR1A3 at different antibody concentrations. Sigmoidal dose–response curves are fitted using Prism Graphpad software (goodness of fit, *R*^2^>0.98).

The binding of uhPR1A3 and ghPR1A3 to SKCO-1, based on FACS analysis, was similar over a wide range of concentrations (0.001–100 *μ*g ml^−1^) (Figure 1; see Supplementary Figure S1). Glycoengineering the Fc segment of PR1A3 has, therefore, no effect on the binding efficiency of the antibody to its epitope on a CEA-expressing CRC line. The affinity of PR1A3 Fab for cell-bound CEA is approximately 10 nM.

**Figure 2:**
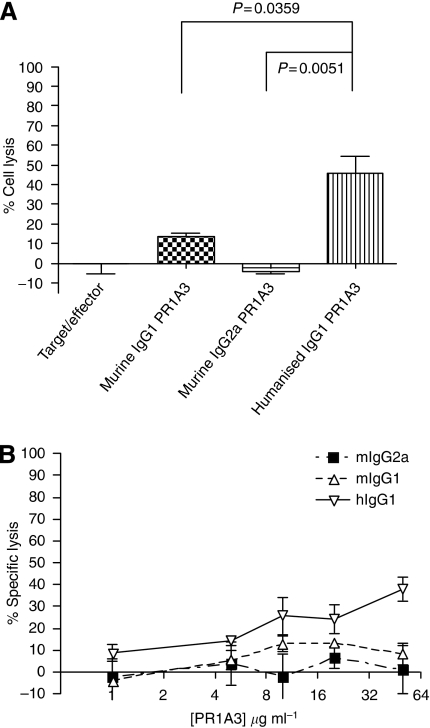
(**A**) Comparison of the ADCC activity of PR1A3 antibodies with differing Fc portions (murine IgG1, murine IgG2a and humanised IgG1). PBMC were used as effectors and SKCO-1 used as the target (100 : 1 effector : target ratio). Antibodies were used at a final concentration of 10 *μ*g ml^−1^. The control was targets and effectors with no antibody. The *P*-values are for the significance of the differences between controls and the hIgG1PR1A3 results, based on *t*-tests. (**B**) Comparison of the ADCC activity of PR1A3 antibodies with differing Fc portions (murine IgG1, murine IgG2a and humanised IgG1) over a range of antibody concentrations from 1 to 50 *μ*g ml^−1^. PBMC were used as effectors and SKCO-1 as target (50 : 1, effector : target ratio).

The ADCC mediated activities of mPR1A3IgG1, mPR1A3IgG2a and uhPR1A3IgG1, using human effector cells and SKCO-1 as targets, are illustrated in Figure 2A and B. The data show that uhPR1A3IgG1 kills much more actively than either mPR1A3IgG1 or mPR1A3IgG2a. PR1A3 does not mediate any killing in the absence of immune cells (data not shown). These data confirm that hPR1A3 mediated killing depends on the appropriate Fc–Fc*γ*R interaction.

**Figure 3:**
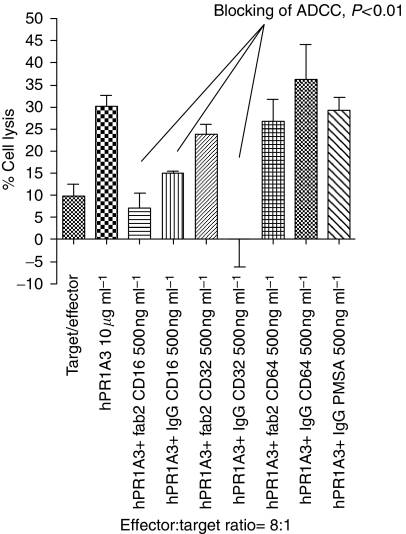
Effect of Fc*γ*R blocking on unmodified humanised PR1A3-induced ADCC, using anti-CD16, 32 and 64 Fab_2_ or IgG. SKCO-1 was used as the target and NK cells as the effectors (effector : target ratio=8 : 1). The *P*-values are for the significance of the differences between the result using hIgG1PR1A3 and no blocking antibody, based on *t*-tests.

Using enriched NK cells as effectors and SKCO-1 as targets, the effects on hPR1A3 mediated ADCC of blocking with Fab_2_ and IgG antibodies against the three known human classes of Fc*γ*Rs, CD64 (Fc*γ*RI), CD32 (Fc*γ*RII) and CD16 (Fc*γ*RIII), are shown in Figure 3. CD16 Fab_2_ blocks the ADCC somewhat more than CD16 IgG, whereas CD32 Fab_2_ had no significant effect. However, CD32 IgG (clone IV.3) was as effective, if not more so, than CD16 IgG in inhibiting both ADCC and antibody-independent killing. Neither CD64 Fab_2_ nor IgG had any significant effect, whereas PMSA antibody, used as a non-specific blocking control, showed the absence of non-specific competition for Fc*γ*Rs on effector cells.

**Figure 4:**
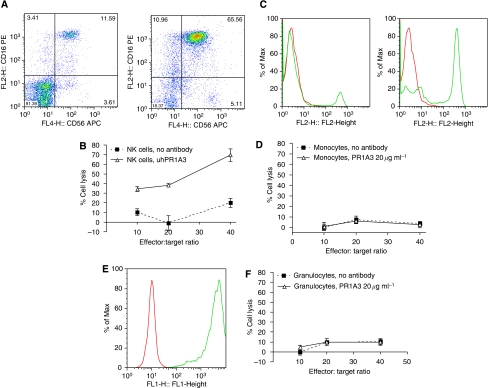
Comparative ADCC activity of different cell types from a single healthy donor. (**A**) FACS analysis dot plot showing the enrichment of NK cells from PBMC (*x*-axis, CD56; *y*-axis, CD16) pre (left-hand dot pot) and post (right-hand dot plot) sorting. (**B**) NK ADCC activity. Graph of NK ADCC activity against SKCO-1 in the absence and presence of 20 *μ*g ml^−1^ of hPR1A3 at different effector : target ratios (*x*-axis) *vs* % cell lysis (*y*-axis). (**C**) FACS analysis plot showing the enrichment of monocytes from PBMC (*x*-axis, CD14 intensity; *y*-axis, number of events)- pre (left-hand histogram plot) and post (right-hand histogram plot) sorting. Grey line presents staining using CD14 antibody-PE; dark line represents staining with isotype antibody control-PE. (**D**) Monocyte ADCC activity. Graph of monocyte ADCC activity against SKCO-1, in the absence and presence of 20 *μ*g ml^−1^ of hPR1A3, at different effector : target ratios (*x*-axis) *vs* % cell lysis (*y*-axis). (**E**) FACS analysis plot showing the enrichment of granulocytes from fresh blood after sorting (*x*-axis, CD15 intensity; *y*-axis, frequency of events). Grey line presents staining using CD15 antibody-FITC; dark line represents staining with isotype antibody control-FITC. (**F**) Granulocyte ADCC activity. Graph of granulocyte ADCC activity against SKCO-1 in the absence and presence of 20 *μ*g ml^−1^ of hPR1A3, at different effector : target ratios (*x*-axis) *vs* % cell lysis (*y*-axis).

NK cells, which predominantly express Fc*γ*RIIIa, mediate ADCC at much lower effector : target ratios than whole PBMC (Schmitz *et al*, 2002; Lazar *et al*, 2006; Conaghan *et al*, 2008). Monocytes, however, express all three classes of Fc*γ*Rs on their surface, and granulocytes express both Fc*γ*RIIa and IIIb. Thus, both these cells types might be expected to contribute to ADCC. NK cells, monocytes and granulocytes were therefore compared as effectors for ADCC using the EuTDA assay, as shown in Figure 4. NK cells, defined as CD3−/CD56+/CD16+, using CD56 (*x*-axis) and CD16 (*y*-axis) antibodies (Figure 4A; left panel before, and right panel after sorting) elicited significant ADCC with 20 *μ*g ml^−1^ of uhPRA1A3 IgG1 and SKCO-1 as targets even at effector to target ratios of 10 : 1 (Figure 4B). Neither, monocytes, defined as CD3−/CD11b+/CD14+/CD15−/CD16low/CD19−/CD32+/CD45+/CD56−/CD64+ (Figure 4C; left panel before, and right panel after sorting using anti CD14 (*x*-axis, stain intensity; *y*-axis, event frequency), nor granulocytes, defined as CD3−/CD14−/CD15+/CD16+/CD19−/CD56− (Figure 4E after enrichment using anti-CD15) showed any evidence of ADCC even at effector : target ratios of up to 40 : 1 (Figure 4D and F).

**Figure 5:**
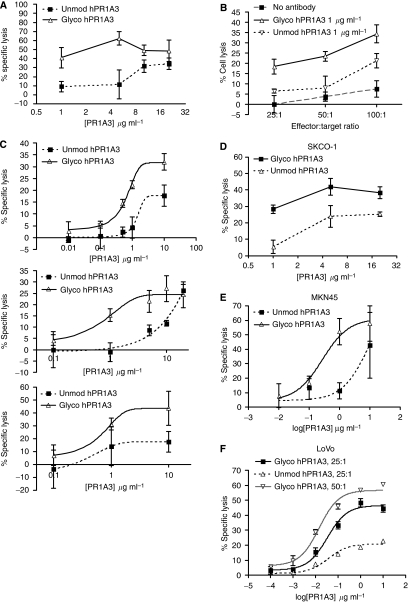
(**A**) Comparison of ADCC activity of glycoform engineered humanised PR1A3 (ghPR1A3) with unmodified humanised PR1A3 (uhPR1A3) using PBMC. SKCO-1 were used as targets. Effector : target ratio used was 50 : 1 (*x*-axis, concentration of PR1A3 used; *y*-axis, % specific lysis). (**B**) Comparison of ADCC activity of ghPR1A3 with uhPR1A3 at different effector : target ratios and a fixed antibody concentration of 1 *μ*g ml^−1^ for both variants. The target cells were SKCO-1 and effectors PBMCs from fresh blood (*x*-axis, effector : target ratio used; *y*-axis, % specific lysis). (**C**) Comparison of ADCC activity of ghPR1A3 with uhPR1A3, using PBMC from three separate donors. SKCO-1 (a high CEA expressing cell line) were the targets. Effector : target ratio was 50 : 1 (*x*-axis, concentration of PR1A3 used; *y*-axis, % specific lysis). (**D**) Comparison of ADCC activity of ghPR1A3 with uhPR1A3 using human NK cells and SKCO-1 as targets (*x*-axis, concentration of PR1A3 used; *y*-axis, % specific lysis). Effector : target ratio used was 10 : 1. (**E**) Comparison of ADCC activity of ghPR1A3 with uhPR1A3, using PBMC as effectors, on MKN45 (an intermediate CEA expressing cell line) as the targets. Effector : target ratio was 50 : 1 (*x*-axis, log concentration of PR1A3 used; *y*-axis, % specific lysis). (**F**) Comparison of ADCC activity of ghPR1A3 with uhPR1A3, using PBMC as effectors on LoVo (an intermediate CEA expressing cell line) as the targets. Effector : target ratio was either 25 : 1 or 50 : 1 (*x*-axis, log concentration of PR1A3 used; *y*-axis, % specific lysis).

Figure 5A shows that glycoengineered antibody, ghPR1A3, elicits ADCC at much lower concentrations than the unmodified form. Thus, just 1 *μ*g ml^−1^ of ghPR1A3 kills over 40% of SKCO-1 target cells, using unfractionated PBMC as effectors, whereas 1 *μ*g ml^−1^ of uhPR1A3 kills only about 10% of the targets, suggesting 10–100-fold increased effectiveness of the glycoengineered antibody. Figure 5B shows that 1 *μ*g ml^−1^ of ghPR1A3 is significantly more effective at mediating killing than is 1 *μ*g ml^−1^ uhPR1A3 over a range of effector to target ratios from 25 : 1 to 100 : 1, again using PBMC as effectors. Figure 5C shows that the difference between the killing efficiency of ghPR1A3 and uhPR1A3 is similar using effector cells from three different PBMC donors whereas Figure 5D shows that using enriched human NK cells as effectors, ghPR1A3 is significantly more effective than uhPR1A3 at killing SKCO-1 cells over a range of concentrations (1–20 *μ*g ml^−1^), as expected if NK cells are the main cell type mediating ADCC in PBMC. Figure 5E and F shows that ghPR1A3 was also significantly more effective than uhPR1A3 in killing the intermediate CEA-expressing cancer cell lines MKN45, and, particularly, LoVo, the latter even at an effector : target ratio of 25 : 1. As expected, no enhancement was seen for HCT116, a cell line that does not express CEA (data not shown).

**Figure 6:**
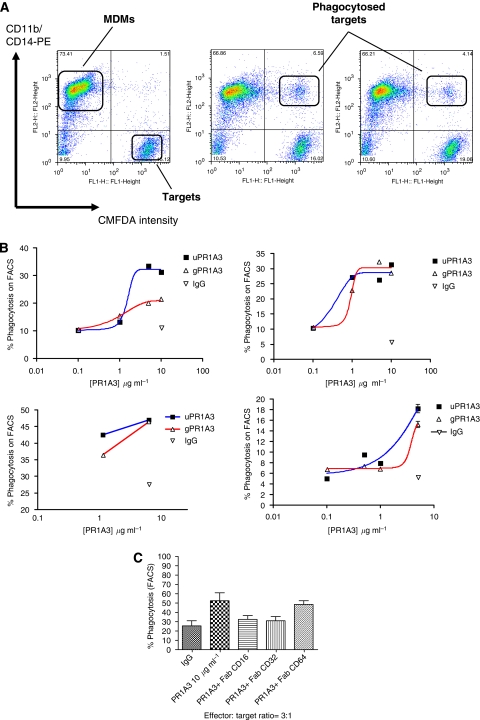

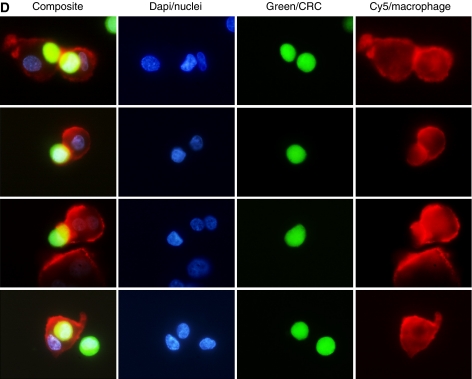
(**A**) Flow cytometric analysis of ADCP. SKCO-1 target cells were stained green with CMFDA and are present in the right lower quadrant of the dot plots. Macrophages were stained with anti-CD11b and CD14 conjugated with PE. They appear in the left upper quadrant of the dot plots. The left hand dot plot is from a representative 1-h culture of macrophages and target cells (SKCO-1) in the presence of IgG isotype control. The middle and right plots are from representative 1-h cultures of macrophages and target cells in the presence of uhPR1A3 and ghPR1A3, respectively (5 *μ*g ml^−1^). The effector : target ratio used was 5 : 1. (**B**) Effect of increasing concentrations of uhPR1A3 and ghPR1A3 on phagocytosis. Tumour targets were pre-incubated with an isotype control antibody (IgG, 10 *μ*g ml^−1^) or the variants of hPR1A3 at concentrations of 0.1–10 *μ*g ml^−1^. ADCP was determined by flow cytometric analysis as the percentage of targets in the upper right hand quadrant (see Figure 6C). The four graphs represent responses from four separate donors. (**C**) Effect of Fc*γ*R blocking on ADCP. Flow cytometry was used to calculate the percentage of tumour cell engulfment by cultured macrophages in the presence of 10 *μ*g ml^−1^ of uhPR1A3. Fab_2_ fragments were used to block either Fc*γ*R I (CD64), Fc*γ*R II (CD32) or Fc*γ*R III (CD16) (each antibody concentration was1 *μ*g ml^−1^). The effector : target ratio used was 3 : 1, and the targets were SKCO-1. (**D**) Fluorescent images of macrophages phagocytosing SKCO-1 using the Ikoniscope. The macrophages (red) have been stained with anti-CD14 and anti-CD11b primary antibodies followed by goat anti-mouse-HRP and Tyramide 647. The target cell line (SKCO-1) was stained green with CMFDA (Celltracker probe). The left hand panels show microscope composite images viewed with FITC (green), Cy5 (for tyramide 647) and DAPI (blue) channels. The second, third and fourth panel column show the same cells viewed separately with the DAPI, green and Cy5 channels. Each represents a different phagocytic event.

To investigate macrophage-based ADCP (Munn *et al*, 1991; Watanabe *et al*, 1999; Lazar *et al*, 2006; McEarchern *et al*, 2007), monocytes were cultured to give rise to macrophages, which by FACS analysis were shown to be negative for CD3, CD15, CD19 and CD56, positive for CD11b, CD14, CD32, CD64 and HLA class II and weakly positive for CD16 (see Supplementary Figure S2a). The cultured monocytes adhere to the bottom of the plates and have an enlarged granular appearance, as expected for macrophages (see Supplementary Figure S2b). Freshly harvested macrophages were incubated with green CMFDA labelled SKCO-1, and a range of antibody concentrations using macrophage : target ratios from 3 : 1 to 8 : 1. A representative FACS analysis of the mixture of macrophages and target SKCO-1 cells after 1-h at 37° in the presence of an isotype control (PMSA), uhPR1A3 and ghPR1A3 antibodies (5.0 *μ*g ml^−1^) is shown in Figure 6A. The green-labelled target cells are in the right lower quadrant and the effectors, labelled with PE conjugated anti-CD11b/CD14, in the left upper quadrant of the dot plots. After one hour's culture in the presence of either uhPR1A3 or ghPR1A3 there were substantial numbers of red and green positive signals in the upper right quadrant, presumably representing macrophages containing engulfed target cells, in contrast to the results for the PMSA control (Figure 6A). The proportion of such signals is essentially the same for both uhPR1A3 and ghPR1A3, and is concentration dependent, as shown in Figure 6B for four different donors. This indicates that, in contrast to ADCC, glycoengineering does not enhance ADCP. The apparent lower level of expression of Fc*γ*RIII (CD16) as compared with Fc*γ*RII (CD32) (see Supplementary Figure S2a) suggests that the density of surface membrane CD16 on macrophages may not be enough for glycoengineering of PR1A3 to enhanced ADCP.

The effects on FACS assayed ADCP of blocking with Fab_2_ fragments each of three classes of Fc*γ*Rs receptor are shown in Figure 6C. Blocking Fc*γ*RII (CD32) or Fc*γ*RIII (CD16) reduced ADCP to some extent, whereas blocking Fc*γ*RI (CD64) had no effect. This indicates that both CD16 and CD32 are involved in ADCP, whereas previously only CD32 was thought to be mainly involved (Richards *et al*, 2008). The CD16 antibody (3G8 clone) used for blocking does not, however, distinguish between Fc*γ*RIIIa and Fc*γ*RIIIb. This suggests that the lack of an effect of glycoengineering on ADCP may be due to a lower concentration of Fc*γ*RIIIa than Fc*γ*RIIIb on macrophages, and the fact that the glycoengineering only enhances the binding to Fc*γ*RIIIa.

For the Ikoniscope-based ADCP assay target, SKCO-1 cells were labelled green with CMFDA and the macrophages red by combining anti-CD11a and anti-CD14 as primary antibodies followed by goat anti-mouse HRP and Tyramide 647 staining. Examples of fluorescent images of macrophages clearly engulfing the target cells, are shown in Figure 6D. In some cases, the macrophages clearly contain two nuclei, one of which is associated with the green stain of a target cell. Using the Ikoniscope it is therefore possible to count, separately, engulfed targets, conjugated targets, free targets and macrophages. Table 1 shows the relative percentage of presumed phagocytosis events seen with the FACS analysis compared with the percentage of confirmed phagocytosis events and percentage of conjugating events seen with the Ikoniscope. Combining engulfed and conjugated targets, as estimated using the Ikoniscope, there is reasonable agreement with the FACS analysis, which cannot distinguish simple attachment from actual engulfment. This suggests that the Ikoniscope-based ADCP assay gives more accurate results than that based on FACS analysis, which is the basis of previous publications reporting ADCP (Munn *et al*, 1991; Huls *et al*, 1999; Akewanlop *et al*, 2001; Lazar *et al*, 2006; McEarchern *et al*, 2007). The quantitative data of Table 1 also suggest that the glycoengineered antibody may be slightly less effective in eliciting ADCP than the unmodified antibody.

**Figure 7:**
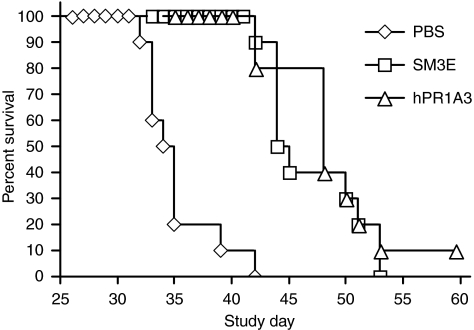
*In vivo* testing of glycoengineered PR1A3. Survival was measured in SCID/beige mice treated with either glycomodified humanised IgG1 PR1A3 (▵), glycomodified IgG1 SM3E (□) or vehicle control (⋄) (*n*=10 in each treatment group). *Y*-axis represents % survival and *x*-axis represents number of days after injection of tumour. Both SM3E and PR1A3 increased survival significantly when compared with the vehicle control (*P*<0.05). There was no significant difference in survival between SM3E and PR1A3 treatments.

To test its *in vivo* efficacy, glycoengineered humanised PR1A3 (IgG1 subclass) was used in a murine CRC model consisting of LS174T induced-liver metastasis. The engineered PR1A3 antibody resulted in significantly improved survival of animals when compared with the vehicle PBS control (see Figure 7). A high affinity IgG1 anti-CEA antibody (SM3E) was also used and was found to prolong survival. This antibody is more effective at eliciting *in vitro* ADCC when compared with PR1A3. However, despite its considerably lower affinity for CEA (*K*_d_, 10 nM *vs* 20 pM), ghPR1A3 was found to be equally effective at prolonging survival when compared with SM3E. These data show that passive immunisation of glycoengineered humanised IgG1 PR1A3 is effective in prolonging survival in a CEA-positive CRC metastatic tumour model.

## Discussion

Antibody engineering to enhance ADCC can be achieved either by altering the amino acid structure of the Fc backbone or by modifying the carbohydrate structures at the hinge region (glycoengineering) to improve the binding affinity to Fc*γ* receptors (Umana *et al*, 1999; Niwa *et al*, 2004b; Lazar *et al*, 2006). Three glycoengineering approaches have been used (i) over expression of the enzyme GntIII in the antibody producing cells, which results in the Fc segment containing an increase in bisected non-fucosylated oligosaccharides, (ii) producing antibody in CHO cells that lack the transferase enzyme involved in core-fucosylation and (iii) using siRNA to knockdown fucosyl transferase activity. Over expression of GntIII has been used successfully to increase the *in vitro* ADCC potency of antibodies IGN311 (Lewis Y-specific) and chCE7 (anti-neuroblastoma) (Umana *et al*, 1999; Schuster *et al*, 2005). Using this technique, we have shown that the ADCC activity of the humanised IgG1 anti-CEA antibody PR1A3 can be increased more than 10-fold. This effect is seen in both intermediate and high expressing CEA cell lines. The level of enhancement may be pronounced even at low effector : target ratios in intermediate expressing CEA cell lines. This underlines the potency of ghPR1A3, which may be particularly relevant considering that antibody penetration into tumours may limit therapeutic capacity. Therefore, it is of paramount importance to show efficient antibody killing by human effector cells. This ADCC activity is still dependent on CD16 (Fc*γ*RIIIa) on the NK cells, as shown by Fab_2_ anti-CD16 blocking and enhanced ADCC of ghPR1A3 over uhPR1A3 in the presence of enriched NK cells. The improved ADCC activity of the glycoengineered antibody is achieved without affecting PR1A3's binding activity, leaving it membrane specific, as described earlier (Durbin *et al*, 1994; Stewart *et al*, 1999). This is an important property of PR1A3, given the finding that soluble CEA can accumulate in lymph nodes and lead to false positive detection of cancers in lymph nodes when using other anti-CEA antibodies for immunoscintigraphy (Granowska *et al*, 1989). In contrast to NK cells, neither freshly isolated granulocytes nor monocytes show any significant ADCC activity at comparable antibody concentrations even with high effector : target ratios. The lack of monocyte-based ADCC activity may be due to the low level of CD16 found in our preparations. We have shown, however, using two different assays, that macrophages kill target tumour cells *in vitro* by ADCP. Both of these mechanisms are therefore likely to have a significant function in *in vivo* patient responses to naked antibody therapy. Glycoengineering substantially improves antibody PR1A3's NK cell mediated ADCC activity, but apparently has no effect on *in vitro* macrophage-based ADCP. This suggests that the Fc*γ*RIIIa receptor is not the effective Fc receptor on macrophages for ADCP and opens up the possibility of alternative engineering of antibodies to enhance their ADCP activity (Richards *et al*, 2008). However, the expression of Fc*γ*Rs on macrophages is dependent on the cytokines in the tumour microenvironment, and so the *in vitro* assay may not reflect the *in vivo* situation, and this needs further investigation. The *in vivo* data presented here show that glycoengineered humanised IgG1 PR1A3 is effective in prolonging survival in a murine CRC metastatic model. This is thought to work by interaction between the Fc segment of the antibody and Fc*γ*RIV, the murine homologue of human Fc*γ*RIIIa. These latter receptors are found on macrophages and granulocytes in mice, emphasising the subtle differences between the mouse and human immune systems. Interestingly, despite the hugely lower affinity for CEA of PR1A3 as compared with SM3E, the improvement in survival was at least as great, if not greater with PR1A3 as with SM3E. This contrasts to the findings *in vitro*, in which glycoengineered SM3E has a much higher ADCC capacity than PR1A3. The *in vivo* finding may be explained by the distinctive specificity of PR1A3 for binding to membrane-bound CEA and scavenging of SM3E by shed CEA. Binding to soluble CEA, leading to sequestration of antibody–antigen complexes away from the tumour, is a major obstacle for any potential therapeutic anti-CEA antibody.

The glycoengineered variant antibodies have been shown to have substantially improved binding to the lower affinity Fc*γ*RIIIa receptor polymorphic variants, which have phenylalanine instead of valine at amino acid position 158 (Ferrara *et al*, 2006). In addition, the carbohydrate residue at Asn-162 of Fc*γ*RIIIa has also been shown to have an important function in the binding of glycoengineered Fc segments, such that its absence leads to greatly attenuated affinity for these antibody variants (Ferrara *et al*, 2006). As only 10–15% of patients have the high affinity Fc*γ*RIIIa receptor polymorphic variants, use of the glycoengineered variant antibody should make this therapy accessible to all patients, whatever their genetic constitution with respect to these Fc*γ*RIIIa receptor polymorphic variants (Cartron *et al*, 2002; Weng and Levy, 2003; Niwa *et al*, 2004a, 2004b; Schuster *et al*, 2005).

Earlier studies on macrophage mediated *in vitro* phagocytosis have relied heavily on FACS analysis using PKH lipid linkers to stain target cells (Munn *et al*, 1991; Huls *et al*, 1999; Akewanlop *et al*, 2001; Lazar *et al*, 2006; McEarchern *et al*, 2007; Richards *et al*, 2008). These linkers can, however, diffuse non-specifically from cell to cell, giving rise to false positive signals and thus limiting their use for phagocytosis assays. The use of a cytoplasmic stain, coupled with the Ikoniscope, shows unequivocally that cultured macrophages are able to phagocytose tumour cells in the presence of PR1A3, and enables satisfactory quantitation of the ADCP assay.

The low relative ADCC activity of the murine IgG2a or IgG1 Fc domains on human cells is consistent with the dependence of ADCC on the specificity of the binding of the Fc portion of an antibody to the relevant Fc receptor (Fc*γ*RIIIa) on NK cells. This may, in part, explain the poor results obtained in early oncology trials using naked murine monoclonal antibodies. On the basis of the blocking experiments with Fab_2_ of CD32 and CD64, these receptors have little if any role in NK mediated ADCC. However, intact anti-CD32 antibody (clone IV.3, IgG2b), in contrast to the Fab_2_ fragment (clone 7.3), is as at least as potent as intact anti-CD16 in inhibiting both ADCC and antibody-independent killing. This may be explained by low levels of CD32c on NK cells (present in 40% of donors; Metes *et al*, 1998) that may lead to self-killing or a secondary cross-linking effect between Fc*γ*Rs.

NK-based ADCC and macrophage-based ADCP are the most likely mechanisms for naked anti-CEA antibody therapy (Munn *et al*, 1991; Huls *et al*, 1999; Watanabe *et al*, 1999; Akewanlop *et al*, 2001; McEarchern *et al*, 2007), as there is no obvious basis for a functional blocking effect involving CEA. In addition, macrophages are known to be potent antigen-presenting cells and so, following antibody stimulated engulfment of whole tumour cells, macrophages may digest the engulfed cells and then re-present peptide fragments to T cells. This may then enable the activation of the adaptive immune system against tumour cell products (Adams and Weiner, 2005; Carter, 2006). It is also likely that dendritic cells, which are super antigen presenters and express Fc*γ*Rs, are stimulated by antibodies to engulf target cells, which would significantly enhance their ability to present tumour antigens to the adaptive immune system (Kalergis and Ravetch, 2002; Benitez-Ribas *et al*, 2006; Melief, 2008). Glycoengineering and other modifications of antibodies to enhance their immune effector functions appear, therefore, to be a most effective way to improve the efficacy of naked antibody therapy by a variety of mechanisms.

## Figures and Tables

**Table 1:** Comparison of the ability of different hPR1A3 variants to elicit ADCP using the Ikoniscope

	**PMSA control**	**ghPR1A3**	**unPR1A3**
Total targets	1336.00	1284.00	1428.00
Targets engulfed by macrophages	27.00	122.00	213.00
Conjugates between targets and macrophages	38.00	79.00	116.00
% of targets engulfed	2.02	9.50	14.92
% of targets conjugated	2.84	6.15	8.12
% phagocytosis+% conjugated	4.87	15.65	23.04
% ADCP using FACS	10.22	22.11	28.56

The number of target cells that were engulfed by macrophages was counted directly using the Ikoniscope. Targets that were conjugated to macrophages were defined as having cell-to-cell membrane contact but no envelopment. Targets and macrophages could readily be identified separately. The percentages of engulfed and conjugated cells are compared with the percentage of targets classified as having undergone ADCP using FACS analysis of the same preparation of cells.

## Supplementary Material

Supplementary Notes
