# Supplementary material for: Humanised IgG1 antibody variants targeting membrane-bound carcinoembryonic antigen by antibody-dependent cellular cytotoxicity and phagocytosis
Source: Br J Cancer. 2009 Nov 10;101(10):1758–68. doi: 10.1038/sj.bjc.6605355 (PMC2778542; doi:10.1038/sj.bjc.6605355)

**Supplementary Notes**

**Methods**

**Isolation of immune effector cells**

**Peripheral blood mononuclear cells (PBMCs)**

PBMCs were isolated either from fresh whole blood from healthy laboratory volunteers, having taken informed consent, or from buffy coats obtained from single donors (*National Blood Service*, Bristol, UK). Following lymphoprep ™ density centrifugation, the layer containing PBMCs was aspirated from the interface and washed with RPMI 1640 to remove excess lymphoprep and platelets [1]. The resulting PBMCs were resuspended complete RPMI 1640, kept at room temperature and used in effector assays within 2 hours.

**NK cell enrichment**.

The *Rosettesep* Human NK isolation cocktail (*Stemcell Technologies*) was used to obtain an enriched population of human natural killer cells from blood, as has been described previously [2]. The enrichment is dependent on a cocktail of antibody tetramers, which recognises a range of proteins on ‘unwanted’ leucocytes. *Rosettesep* cocktail of antibodies was added to blood before lymphoprep™ (*Axis-Shield*) separation. NK cells were aspirated from the interface between plasma and lymphoprep™ and resuspended in 50mls of RPMI 2%/FCS 2% and washed three times. RBC lysis buffer (*BD Pharmingen*) was used if there was excessive red cell contamination. Finally, the cell pellet was re-suspended in RPMI complete medium and counted. An aliquot was taken for flow cytometry analysis which was performed as described previously [3].

**Granulocyte enrichment**

Granulocytes were separated using one-step® Polymorph (*Accurate Chemical & Scientific Corporation*) which contains Sodium Diatrizoate, 13.4% and Dextran 500, 8.0%. Briefly, whole blood was diluted one to one with RPMI 1640 / citrate solution and over-layered on to one-step® Polymorph (specific density of 1.113+/-0.001g/ml). After centrifugation, two leucocyte bands were observed: the top band consisted of mononuclear cells and the bottom layer of polymorphonuclear cells. The lower layer was aspirated and diluted 1:1 with 0.5 normal concentration of culture medium in order to restore the osmolality of the mixture. The cells were washed twice before adding to RPMI complete medium. An aliquot was removed for flow cytometry analysis and tested for CD3, CD14, CD15, CD16 and CD56 expression.

**Monocyte enrichment**

Monocytes were enriched from buffy coats using the *Rosettesep* Human Monocyte Enrichment Cocktail (*Stemcell Technologies*). This works in a similar manner to the NK Enrichment Kit (*Stemcell Technologies*) that is described above. An aliquot was taken for flow cytometry analysis, as described in the paper, and phenotyped for CD3, CD11b, cd14, CD15, CD16, CD19, CD32, CD45, CD56, CD64 and HLA class II (DA-2).

**Culture of Monocyte-derived Macrophages (MDMs) for use in phagocytosis assays**

Enriched monocytes were resuspended at 1.0-1.5x10^6^ cells/ml in X-VIVO 15 medium (*Lonza*) supplemented with 10% FCS, 10ng/ml human M-CSF (*Peprotech*, UK) and 10ng/ml human GM-CSF (*Peprotech*, UK) and cultured on 50mm Lumox Petraperm Plates (*Greiner*) for 8-12 days at 37^o^ in a 5% CO2 incubator. The night before use in an ADCP assay, the medium was changed and X-VIVO 15 medium with 25ng/ml human γIFN and 5ng/ml of human M-CSF was added. The next day the cells were harvested, washed and resuspended in RPMI complete at a concentration of 0.5-2x10^6^ cells/ ml.

Adherent target cells were labelled with 3μM CellTracker Green CMFDA (5-chloromethylfluorescein diacetate), *Invitrogen,* in plain RPMI for 30 minutes at 37^o^C (see supplementary notes for more details). This molecular fluorescent probe works by passing through the cell membrane into the cytoplasm, where it reacts with glutathiones (which is present in cells as protein and non-protein thiol compounds) catalysed by glutathione-S-transferase, resulting in a fluorescent product that is no longer able to pass through the membrane. Free label was washed off using PBSA and the cells were incubated in 10mls of RPMI complete at 37^o^C for 30 minutes, after which they were detached, washed and counted. Labelled target cells were incubated on ice for twenty minutes with varying concentrations of uhPR1A3 or ghPR1A3 (0.1μg-10μg/ml) before 100μl of effectors were added (3:1 to 8:1 effector target ratio). FACS analysis was then performed using the FACS Calibur.

***Ikoniscope***^R^ **imaging system**

This system was developed by Ikonysis, Inc. (New Haven, Connecticut, USA). It contains epifluorescence optics (*Olympus*) and a high resolution monochrome charge coupled device camera (Hamamatsu Orca ER, Hamamatsu Photonic Systems, Bridgewater, NJ). Rapid throughput is enabled by the use of an automated cassette feeder (capacity of 175 slides). Machine manipulation of the slides allows for movement in three planes (x, y and z directions). Combination of automated exposure setting and focusing, together with three-dimensional image acquisition enables the rapid acquisition of high quality images.

**Supplementary Figure Legends**

Figure S1. Comparison of the binding of unmodified humanised IgG1 PR1A3 (uhPR1A3) and glycoengineered IgG1 PR1A3 (ghPR1A3) to the high CEA expressing cell line SKCO-1. FACS staining compares the ability of uhPR1A3 (top row of graphs) with ghPR1A3 (bottom row of graphs) to bind to SKCO-1 at different concentrations of antibody (range 0.001-100μg/ml, from left to right in increasing order). The graphs are plots of the distributions of antibody stained cells (x-axis, intensity of stain on FL-1 channel; y-axis, number of events). The secondary antibody used was anti-human IgG conjugated with FITC.

Figure S2a: FcγR flow cytometry analysis of the surface expression of CD16, CD32, CD64, CD11b, CD14 and HLA Class II (DA-2) (histogram plots: x-axis, intensity of stain, y-axis, frequency of events of cultured monocytes). The green line represents the staining with the respective antibody; the red line represents the staining with an isotype control antibody.

Figure S2b: Morphology of monocytes after 10 days in culture. Macrophages were derived from monocytes by culture on Lumox Petraperm plates in X-VIVO medium supplemented with 10% FCS and human recombinant M-CSF/GM-CSF/γIFN. Photomicrograph at 40x magnification.

**References**

1. Boyum, A. (1968). Separation of leukocytes from blood and bone marrow. Introduction. Scand J Clin Lab Invest Suppl *97*, 7.

2. Roda, J.M., Parihar, R., Lehman, A., Mani, A., Tridandapani, S., and Carson, W.E., 3rd (2006). Interleukin-21 enhances NK cell activation in response to antibody-coated targets. J Immunol *177*, 120-129.

3. Conaghan, P.J., Ashraf, S.Q., Tytherleigh, M.G., Wilding, J.L., Tchilian, E., Bicknell, D., Mortensen, N.J., and Bodmer, W.F. (2008). Targeted killing of colorectal cancer cell lines by a humanised IgG1 monoclonal antibody that binds to membrane-bound carcinoembryonic antigen. Br J Cancer *98*, 1217-1225.

**Figure S1.**


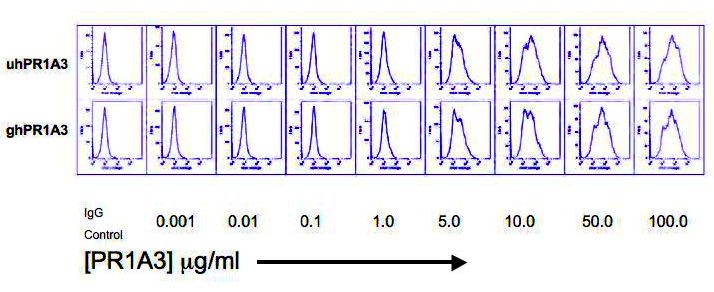


**Figure S2a:**


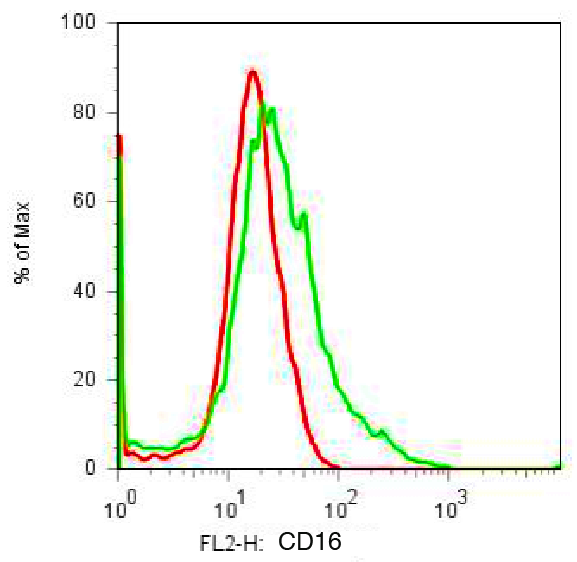

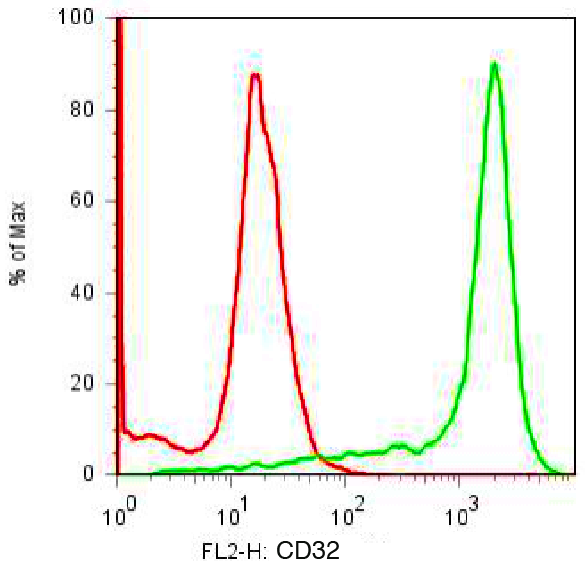

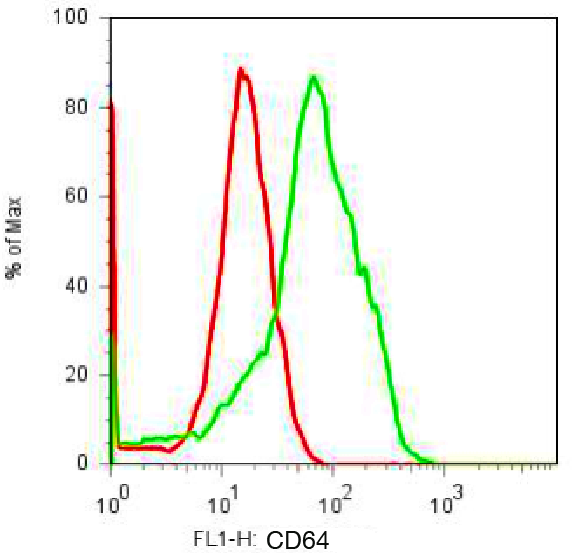


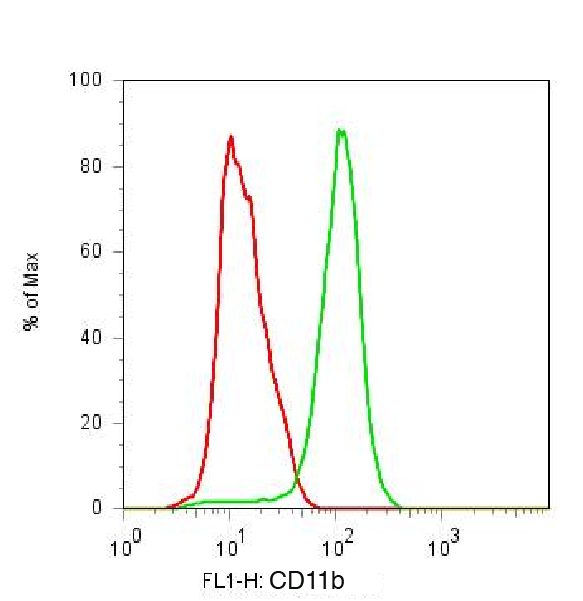

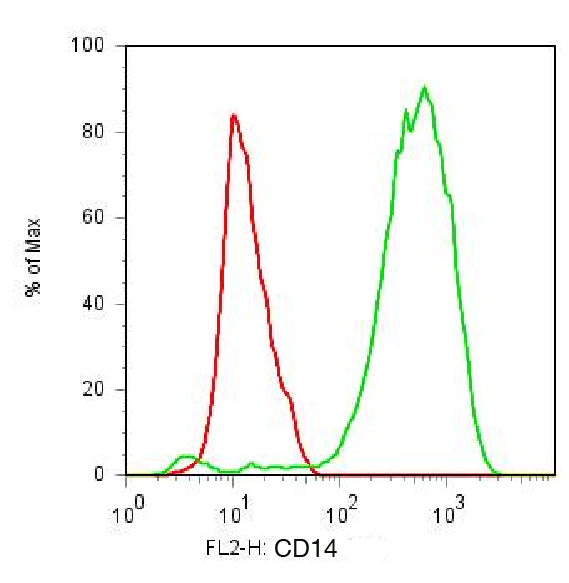

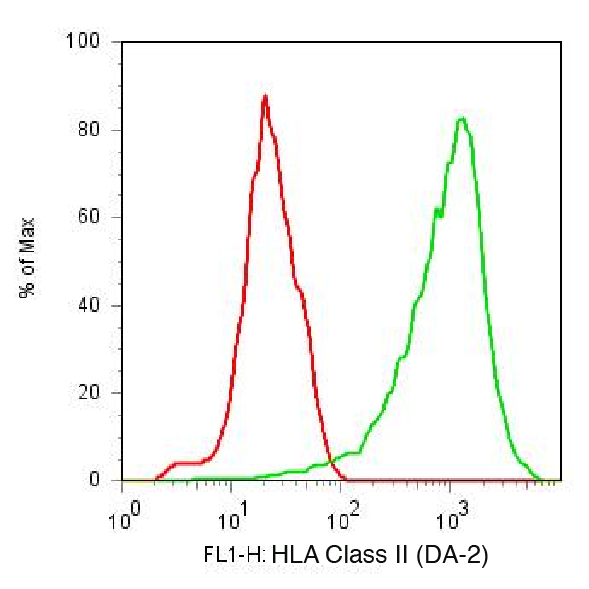


**Figure S2b:**


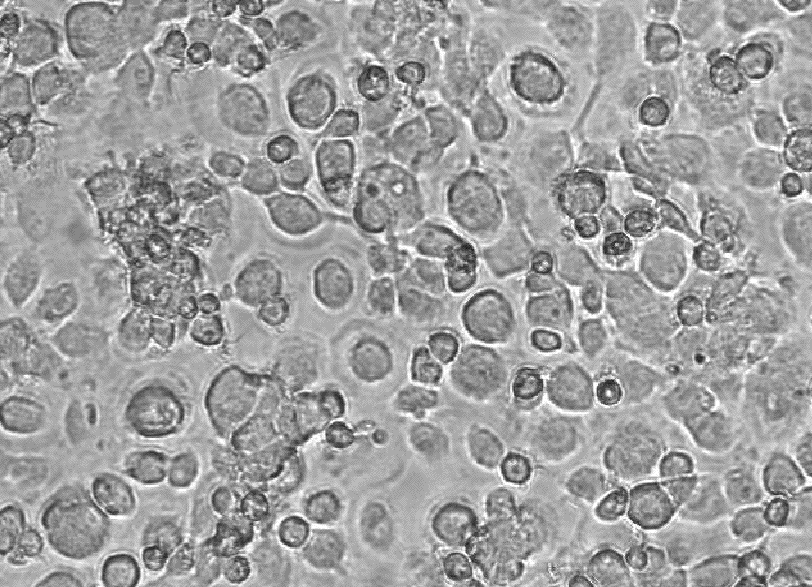

Supplement: Supplementary Notes [file 6605355x1.doc]
